# The Advisory Committee on Immunization Practices’ Interim Recommendation for Allocating Initial Supplies of COVID-19 Vaccine — United States, 2020

**DOI:** 10.15585/mmwr.mm6949e1

**Published:** 2020-12-11

**Authors:** Kathleen Dooling, Nancy McClung, Mary Chamberland, Mona Marin,, Megan Wallace, Beth P. Bell, Grace M. Lee, H. Keipp Talbot, José R. Romero, Sara E. Oliver

**Affiliations:** ^1^CDC COVID-19 Response Team; ^2^General Dynamics Information Technology, Falls Church, Virginia; ^3^Epidemic Intelligence Service, CDC; ^4^University of Washington, Seattle, Washington; ^5^Stanford University School of Medicine, Stanford, California; ^6^Vanderbilt University School of Medicine, Nashville, Tennessee; ^7^Arkansas Department of Health, Little Rock, Arkansas.

The emergence of SARS-CoV-2, the virus that causes coronavirus disease 2019 (COVID-19), has led to a global pandemic that has disrupted all sectors of society. Less than 1 year after the SARS-CoV-2 genome was first sequenced, an application* for Emergency Use Authorization for a candidate vaccine has been filed with the Food and Drug Administration (FDA). However, even if one or more vaccine candidates receive authorization for emergency use, demand for COVID-19 vaccine is expected to exceed supply during the first months of the national vaccination program. The Advisory Committee on Immunization Practices (ACIP) advises CDC on population groups and circumstances for vaccine use.^†^ ACIP convened on December 1, 2020, in advance of the completion of FDA’s review of the Emergency Use Authorization application, to provide interim guidance to federal, state, and local jurisdictions on allocation of initial doses of COVID-19 vaccine. ACIP recommended that, when a COVID-19 vaccine is authorized by FDA and recommended by ACIP, both 1) health care personnel^§^ and 2) residents of long-term care facilities (LTCFs)^¶^ be offered vaccination in the initial phase of the COVID-19 vaccination program (Phase 1a**).^††^ In its deliberations, ACIP considered scientific evidence of SARS-CoV-2 epidemiology, vaccination program implementation, and ethical principles.^§§^ The interim recommendation might be updated over the coming weeks based on additional safety and efficacy data from phase III clinical trials and conditions of FDA Emergency Use Authorization.

Evidence-based information addressing COVID-19 vaccine topics including early allocation has been explicitly and transparently reviewed during seven public ACIP meetings (*1*). To inform policy options for ACIP, the COVID-19 Vaccines Work Group, comprising experts in vaccines and ethics, held more than 25 meetings to review data regarding vaccine candidates, COVID-19 surveillance, and modeling, as well as the vaccine allocation literature from published and external expert committee reports.

Health care settings in general, and long-term care settings in particular, can be high-risk locations for SARS-CoV-2 exposure and transmission (*2*–*4*). Health care personnel are defined as paid and unpaid persons serving in health care settings who have the potential for direct or indirect exposure to patients or infectious materials. As of December 1, 2020, approximately 245,000 COVID-19 cases and 858 COVID-19-associated deaths had been reported among U.S. health care personnel (*5*). Early protection of health care personnel is critical to preserve capacity to care for patients with COVID-19 or other illnesses. LTCF residents are defined as adults who reside in facilities that provide a range of services, including medical and personal care, to persons who are unable to live independently. LTCF residents, because of their age, high rates of underlying medical conditions, and congregate living situation, are at high risk for infection and severe illness from COVID-19. As of November 15, 2020, approximately 500,000 COVID-19 cases and 70,000 associated deaths had been reported among residents of skilled nursing facilities, a subset of LTCFs serving residents with more complex medical needs (*6*).

With respect to vaccination program implementation, vaccines that require cold and ultracold storage, specialized handling, and large minimum order requirements are most feasibly maintained in centralized vaccination clinics, such as acute health care settings, or through the federal Pharmacy Partnership for Long-term Care Program.^¶¶^ ACIP’s ethical principles for allocating initial supplies of COVID-19 vaccine, namely to maximize benefits and minimize harms, promote justice, and mitigate health inequities (*7*), support the early vaccination of health care personnel and LTCF residents.

Approximately 21 million U.S. health care personnel work in settings such as hospitals, LTCFs, outpatient clinics, home health care, public health clinical services, emergency medical services, and pharmacies. Health care personnel comprise clinical staff members, including nursing or medical assistants and support staff members (e.g., those who work in food, environmental, and administrative services) (*8*). Jurisdictions might consider first offering vaccine to health care personnel whose duties require proximity (within 6 feet) to other persons. If vaccine supply remains constrained, additional factors might be considered for subprioritization.*** Public health authorities and health care systems should work together to ensure COVID-19 vaccine access to health care personnel who are not affiliated with hospitals.

Approximately 3 million adults reside in LTCFs, which include skilled nursing facilities, nursing homes, and assisted living facilities. Depending upon the number of initial vaccine doses available, jurisdictions might consider first offering vaccination to residents and health care personnel in skilled nursing facilities because of high medical acuity and COVID-19–associated mortality (*6*) among residents in these settings.

Monitoring vaccine safety in all populations receiving COVID-19 vaccine is required under an Emergency Use Authorization. Vaccines are being studied in older adults with underlying health conditions; however, LTCF residents have not been specifically studied. ACIP members called for additional active safety monitoring in LTCFs to ensure timely reporting and evaluation of adverse events after immunization. ACIP will consider vaccine-specific recommendations and additional populations for vaccine allocation beyond Phase 1a when an FDA-authorized vaccine is available.

**Summary**

**What is already known about this topic?**

Demand is expected to exceed supply during the first months of the national COVID-19 vaccination program.

**What is added by this report?**

The Advisory Committee on Immunization Practices (ACIP) recommended, as interim guidance, that both 1) health care personnel and 2) residents of long-term care facilities be offered COVID-19 vaccine in the initial phase of the vaccination program.

**What are the implications for public health practice?**

Federal, state, and local jurisdictions should use this guidance for COVID-19 vaccination program planning and implementation. ACIP will consider vaccine-specific recommendations and additional populations when a Food and Drug Administration–authorized vaccine is available.

## Footnotes

* https://www.pfizer.com/news/press-release/press-release-detail/pfizer-and-biontech-submit-emergency-use-authorization.

† https://www.cdc.gov/vaccines/acip/committee/acip-charter.pdf.

§ https://www.cdc.gov/infectioncontrol/guidelines/healthcare-personnel/appendix/terminology.html.

¶ https://www.cdc.gov/longtermcare/index.html.

**
https://www.cdc.gov/vaccines/acip/meetings/slides-2020-11.html .

†† On December 1, 2020, ACIP voted 13-1 in favor of the Phase 1a allocation recommendation.

§§ https://www.cdc.gov/vaccines/hcp/acip-recs/vacc-specific/covid-19/evidence-table.html.

¶¶ https://www.cdc.gov/vaccines/covid-19/long-term-care/pharmacy-partnerships.html.

*** https://www.cdc.gov/vaccines/hcp/acip-recs/vacc-specific/covid-19/clinical-considerations.html.
